# Optimization of Electrospinning Parameters for Lower Molecular Weight Polymers: A Case Study on Polyvinylpyrrolidone

**DOI:** 10.3390/polym16091217

**Published:** 2024-04-26

**Authors:** Fatima Tuz Zahra, Ying Zhang, Adeolu Oluwaseun Ajayi, Quincy Quick, Richard Mu

**Affiliations:** 1 TIGER Institute, Tennessee State University, Nashville, TN 37209, USA; 2 Center for Manufacturing Research, Tennessee Technological University, Cookeville, TN 38505, USA; yzhang@tntech.edu; 3 Department of Industrial Engineering and Operations Research, Columbia University in the City of New York, New York, NY 10027, USA; aoa2160@columbia.edu; 4 Department of Biological Sciences, Tennessee State University, Nashville, TN 37209, USA; qquick@tnstate.edu

**Keywords:** Polyvinylpyrrolidone, low molecular weight, electrospinning, nanofibers, parameters optimization, quantitative analysis, morphology

## Abstract

Polyvinylpyrrolidone (PVP) is a synthetic polymer that holds significance in various fields such as biomedical, medical, and electronics, due to its biocompatibility and exceptional dielectric properties. Electrospinning is the most commonly used tool to fabricate fibers because of its convenience and the wide choice of parameter optimization. Various parameters, including solution molarity, flow rate, voltage, needle gauge, and needle-to-collector distance, can be optimized to obtain the desired morphology of the fibers. Although PVP is commercially available in various molecular weights, PVP with a molecular weight of 130,000 g/mol is generally considered to be the easiest PVP to fabricate fibers with minimal challenges. However, the fiber diameter in this case is usually in the micron regime, which limits the utilization of PVP fibers in fields that require fiber diameters in the nano regime. Generally, PVP with a lower molecular weight, such as 10,000 g/mol and 55,000 g/mol, is known to present challenges in fiber preparation. In the current study, parameter optimization for PVP possessing molecular weights of 10,000 g/mol and 55,000 g/mol was carried out to obtain nanofibers. The electrospinning technique was utilized for fiber fabrication by optimizing the above-mentioned parameters. SEM analysis was performed to analyze the fiber morphology, and quantitative analysis was performed to correlate the effect of parameters on the fiber morphology. This research study will lead to various applications, such as drug encapsulation for sustained drug release and nanoparticles/nanotubes encapsulation for microwave absorption applications.

## 1. Introduction

**Figure 1:**
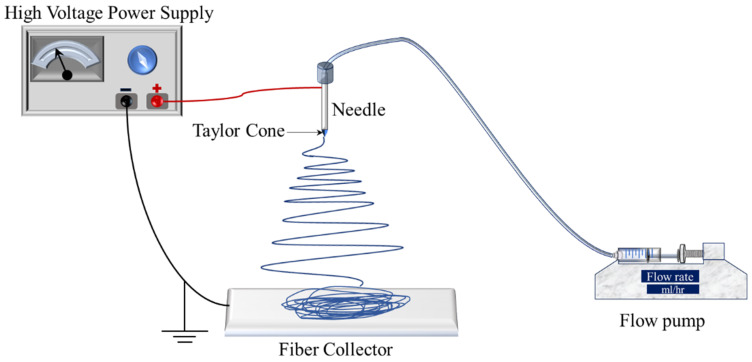
Schematic of electrospinning process.

The electrospinning technique is a versatile method for fabricating fibers from a broad range of polymers. This process offers a wide array of controllable parameters, including flow rate (measured in mL/h), applied voltage (kV), needle gauge (G), and the distance between the needle tip and the collector (cm), as depicted in Figure 1’s schematic of the electrospinning setup.

To begin electrospinning, a polymer solution is loaded into a syringe (connected to a metal needle), which is then set to a specific flow rate. The needle is connected to a direct current (DC) voltage supply that applies a high voltage, while the collector is grounded at a designated distance from the needle tip. Upon applying the voltage, a phenomenon to form the Taylor cone occurs, generating a fine fiber jet that propels toward the grounded collector. The careful manipulation of these process parameters allows for the customization of fiber morphology to meet specific application needs. The formation of the Taylor cone is crucial for successful fiber production. Therefore, optimizing the electrospinning parameters, in conjunction with the intrinsic properties of the polymer solution—such as its volatility, viscosity, and conductivity—is essential. These properties not only influence the ability to form a Taylor cone but also affect the fiber morphology [1].

Ambient conditions, including temperature and humidity, also play a significant role in determining fiber morphology. The choice of solvent for the polymer solution impacts its volatility; this is particularly important in biomedical applications like drug delivery, where a non-volatile solution may be detrimental [2,3,4]. Additionally, the molecular weight (Mw) of the polymer and the concentration of the polymer in the solution are key factors that govern the degree of polymer chain entanglement, which in turn affects the solution’s viscosity and the quality of the electrospun fibers [5].

The fabrication of fibers relies on the equilibrium of forces resulting from surface tension, the concentration of charges on the jet, and the viscosity of the solution. The viscosity (*η*) and molecular weight of the polymer are directly related, and their relationship is expressed in the form of the Mark–Houwink Equation [6,7]

$$\left[\eta \right]=K\cdot M_{v}^{\alpha }$$

where α and *K* are constants for a given polymer, solvent, and temperature.

Surface tension causes the liquid jet to transform into one or multiple spherical droplets in order to minimize surface area due to Rayleigh instability [8]. On the other hand, the electrostatic repulsion between charges on the jet counteracts this effect and promotes an increase in surface area, leading to the creation of a thin jet. The viscoelastic forces present in a polymer solution also play a vital role in bead-free fiber fabrication, as they prevent changes in shape and help create fibers. By adjusting the interplay between these forces, various fiber structures can be produced. For instance, by strengthening the impact of viscoelastic and charge repulsion forces compared to surface tension, it is possible to prevent the formation of beads. While the impact of solution concentration on fiber structure is widely known, it is the alteration in solution viscosity that ultimately leads to changes in the morphology of electrospun fibers [9].

Electrospinning fibers from polymers with lower molecular weights (M_w_) presents significant challenges due to the inherent physical properties of these materials. High-molecular-weight polymers are typically favored in electrospinning because they offer the necessary chain entanglement that facilitates fiber formation. In contrast, when using polymers with lower M_w_, the surface tension of the polymer solution often exceeds its viscoelastic forces. This imbalance results in the formation of beads along the fibers, which is an undesirable outcome. Beads within the fiber structure can significantly impair the mechanical integrity and functional performance of the resultant fiber mat, as they act as sites of structural weakness and/or defects. To mitigate this issue and achieve a uniform, beadless fiber morphology, careful optimization of electrospinning parameters is essential. This includes adjusting the solution concentration, as well as the electrospinning voltage, flow rate, and distance between the needle and collector. Achieving the optimal set of conditions is crucial to produce defect-free electrospun fibers, which is vital for their application in fields where high performance and consistency of physical properties are imperative.

**Figure 2:**
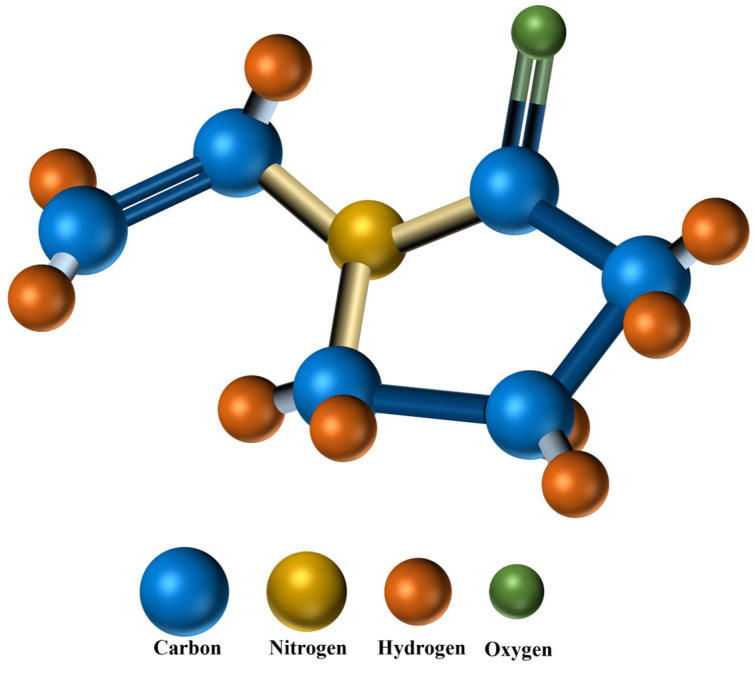
Ball-and-stick model of PVP molecular structure.

Polyvinylpyrrolidone (PVP) is a versatile synthetic polymer, denoted by the molecular formula (C_6_H_9_NO)_n_. The ball-and-stick model presented in Figure 2 illustrates the molecular composition of PVP. This polymer has been extensively combined with materials such as titanium dioxide (TiO_2_), Titanium nitride (TiN), and carbon nanotubes (CNTs) to explore its potential in sensing technologies [10,11,12]. The biocompatibility and nontoxicity of PVP also make it a popular choice in the field of drug delivery systems [13,14].

Commercially, PVP is available in a spectrum of molecular weights (M_w_), but it is commonly utilized at M_w_ ≥ 130,000 g/mol for various applications. Recent research has highlighted how different electrospinning parameters affect the morphology of PVP fibers, with these studies typically using a molecular weight of 130,000 g/mol as a standard [15,16,17,18,19]. However, there is a noticeable gap in the literature regarding the systematic exploration of electrospun PVP fibers at lower molecular weights.

Addressing this research gap could reveal significant insights into the fundamentals of electrospinning PVP. By tailoring the solution concentration (expressed in weight percent, wt%) for lower molecular weights, it may be possible to produce thinner fibers. These finer fibers can have the potential to enhance performance across a range of applications due to their increased surface area and unique physical properties. Therefore, further investigation into the optimization of electrospinning parameters for PVP with lower molecular weights is not only warranted but could also open new avenues for material innovation.

In bridging the current knowledge gap, this study offers a significant exploration of the electrospinning process using lower molecular weight Polyvinylpyrrolidone (PVP) variants, specifically 10,000 and 55,000 g/mol. Through careful optimization of the solution concentration (wt%) and electrospinning parameters, we have successfully fabricated uniform electrospun fibers. Additionally, this study provides insight into the effects of needle gauge size and the distance from the needle tip to the collector (NCD) on the fiber morphology. Our findings elucidate crucial electrospinning process parameters that markedly influence fiber fabrication, particularly for polymers with lower molecular weights. This research not only advances our fundamental understanding of electrospinning dynamics but also paves the way for innovative applications of fine-tuned nanofibrous materials.

## 2. Experimental Details

### 2.1. Materials

Polyvinylpyrrolidone (PVP) possessing M_w_ 10,000 g/mol and 55,000 g/mol, as well as solvent Ethanol (purity 99.9%), were purchased from Sigma Aldrich (St. Louis, MO, USA) to carry out the experimental work.

### 2.2. Parameter Optimization for PVP (M_w_~10,000 g/mol and 55,000 g/mol)

Polyvinylpyrrolidone (PVP) exhibits solubility in a wide range of organic solvents as well as deionized water. In this study, ethanol was employed as a solvent for the preparation of the PVP polymer solution. This choice was made due to the higher evaporation rate of the PVP solution at ambient temperature. Polymer solutions of PVP with varying molecular weights were formulated at concentrations of 10 wt%, 30 wt%, and 50 wt%. It is noteworthy that wt% denotes the ratio of the mass of PVP to the mass of ethanol × 100. To ensure uniformity, the solutions underwent magnetic stirring for one hour at room temperature. Subsequently, the solutions were loaded into a 10 mL syringe connected to a flow meter/syringe pump (1000-US/SyringeONE, New Era Pump Systems Inc., Farmingdale, NY, USA) for the electrospinning process. The process parameters utilized for electrospinning are detailed in Table 1.

### 2.3. Effect of Variation in Needle Gauge and Needle Tip-to-Collector Distance

In this phase of the experimental investigation, a consistent flow rate of 0.5 mL/h and an applied voltage of 20 kV were maintained for all samples. The 25G needle, with an inner diameter (ID) of 0.260 mm, was substituted with a 22G needle (ID~0.413 mm), while the distance between the needle and collector was adjusted within the range of 12–16 cm. Spinning solutions of 80 wt% PVP_10000_ and 50 wt% PVP_55000_ were employed for the experimental procedures. The deposition duration for each sample was controlled within an interval of 5–10 min. Electrospinning parameters used in this part of the experimental work are tabulated in Table 2.

### 2.4. Characterization

The morphology of the samples was analyzed using scanning electron microscopy (Zeiss Merlin). ImageJ software (https://imagej.net/ij/) was utilized to estimate the fiber diameter from SEM images, with 40 fibers examined for each sample to investigate the impact of process parameters on morphology. The diameter distribution of all samples was further analyzed using OriginPro 2024.

## 3. Results and Discussion

The results section will be subdivided into three parts, discussing the parameter optimization for PVP_10000_ and PVP_55000_, as well as the effect of variation in needle gauge and NCD.

### 3.1. Parameter Optimization for PVP_10000_

In the electrospinning experiments conducted at room temperature, three PVP_10000_ solutions with concentrations of 10 wt%, 30 wt%, and 50 wt% were utilized, adhering to the process parameters delineated in the experimental section. Subsequent SEM analysis was performed on the derived samples. As depicted in Figure S1, the samples generated from the 10 wt% solution did not yield fibers; instead, particle formation was observed across all tested voltages and flow rates. This outcome is attributed to the low polymer concentration, which is hypothesized to increase the surface tension and reduce the solution’s viscosity, thereby inhibiting the elongation necessary for fiber formation. Further investigation into the relationship between polymer concentration, surface tension, and viscosity is warranted to substantiate this hypothesis and refine the electrospinning process parameters for low-concentration solutions.

**Figure 3:**
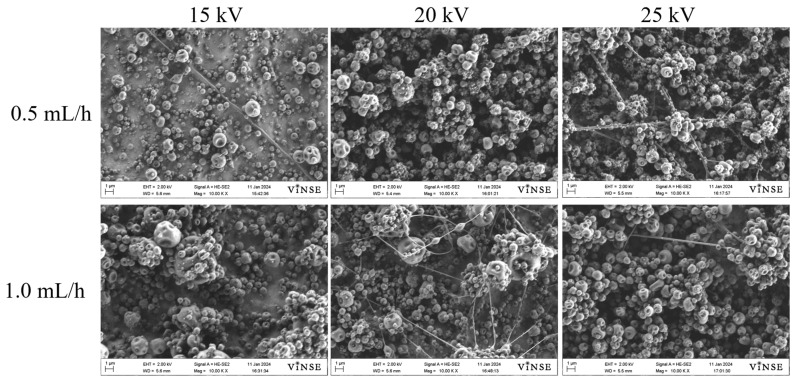
SEM micrographs of samples prepared using 30 wt% PVP_10000_ solution at 14 cm NCD.

**Figure 4:**
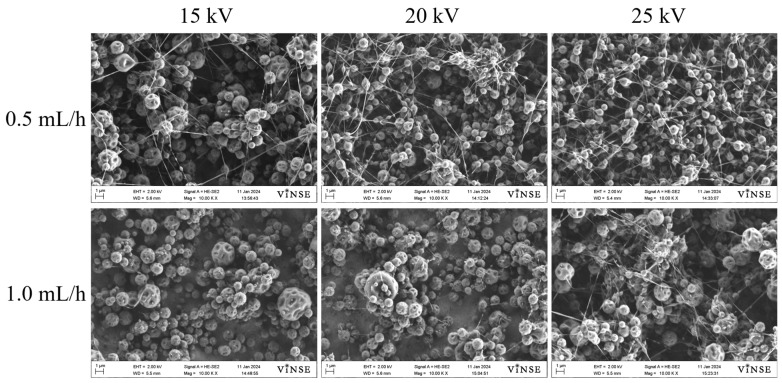
SEM micrographs of samples prepared using 50 wt% PVP_10000_ solution at 14 cm NCD.

The concentration of PVP_10000_ was increased to 30 wt% and 50 wt%. While fibers tended to form, particle formation was significantly higher than fibers. These results indicate that increasing the polymer concentration leads to an increase in the viscosity and viscoelasticity of the solution. This increase overcomes the surface tension of the solution, allowing fiber fabrication to occur. SEM micrographs of these samples are shown in Figure 3 and Figure 4.

Higher concentrations of PVP_10000_ increased the viscosity of the spinning solution, but the spinnability remained low. In the 30 wt% solution, fibers tended to form at a higher flow rate of 1.0 mL/h at 20 kV (Figure 3). However, with the 50 wt% spinning solution, a lower flow rate of 0.5 mL/h showed a higher fiber density (Figure 4).

**Figure 5:**
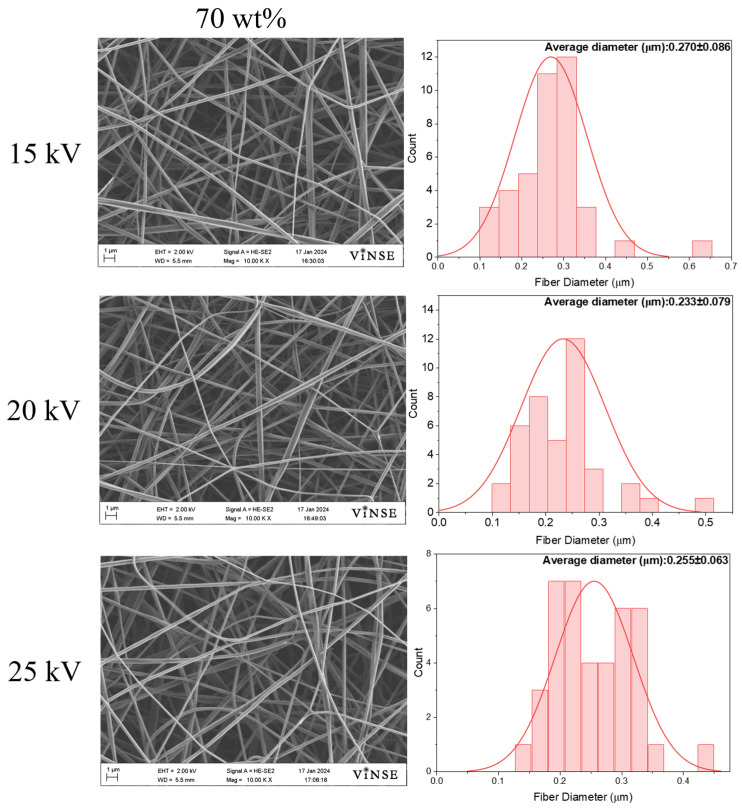
SEM micrographs and fiber diameter distribution graphs of samples prepared with 70 wt% PVP_10000_ solution at 14 cm NCD and 0.5 mL/h flow rate.

Particle density remained high for all voltages at both flow rates, prompting an increase in PVP_10000_ concentration to 70 wt%. Figure 5 illustrates SEM micrographs and their respective fiber diameter distribution curves.

Considering the increase in fiber density at a lower flow rate for the 50 wt% spinning solution, electrospinning of the 70 wt% spinning solution was conducted at 0.5 mL/h, with other process parameters unchanged. This solution exhibited a higher fiber density and lower bead density. Increasing the voltage reduced bead formation, but it was not raised above 25 kV to prevent instability of the Taylor cone [20] and to ensure a uniform fiber diameter distribution. Analysis of fiber diameter distribution revealed that higher voltages resulted in smaller average fiber diameters. The average diameter of all samples was below 0.300 µm, indicating enhanced uniformity with increasing voltage. The better uniformity of the fiber diameter depicted the stabilization of the Taylor cone at higher voltages, which led to uniform stretching of the fiber jet. Average diameters were observed to be 0.270 ± 0.086 µm, 0.233 ± 0.079 µm, and 0.255 ± 0.063 µm for 15 kV, 20 kV, and 25 kV applied voltages, respectively. The decrease in fiber diameter at 20 kV suggests that the higher charge density assisted in stretching the fiber jet. However, at 25 kV, the fiber diameter tends to increase, which is attributed to higher solution ejection from the needle tip due to increased applied voltage [21]. No particle formation was observed for these samples at any voltage. Moreover, the higher PVP_10000_ concentration led to increased viscoelasticity and spinnability of the solution.

**Figure 6:**
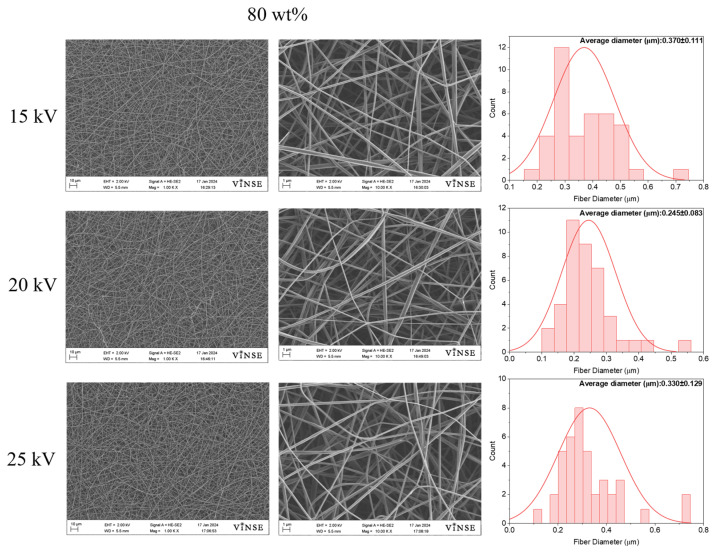
SEM micrographs and fiber diameter distribution graphs of samples prepared with 80 wt% PVP_10000_ solution at 14 cm NCD and 0.5 mL/h flowrate.

To completely avoid bead formation, the PVP_10000_ concentration was increased to 80 wt%. SEM results revealed no bead formation for any of these samples (Figure 6).

This solution concentration (80 wt%) was opted to be the optimized wt% for PVP_10000_. The surface tension was completely overcome by the viscoelastic forces, which as a result produced fibers with fine morphology and no bead formation [22]. The fiber diameter was observed to decrease at 20 kV as compared to 15 kV; however, at 25 kV, it tended to increase. The increase in the average fiber diameter at a higher voltage is attributed to an increase in polymer solution extraction. It is worth mentioning that the increase in concentration from 70 wt% to 80 wt% led to an increase in the average fiber diameters at all applied voltages. Average diameters were 0.370 ± 0.111 µm, 0.245 ± 0.083 µm, and 0.330 ± 0.129 µm for 15 kV, 20 kV, and 25 kV. The diameter distribution was observed to be uniform at 20 kV for both 70 wt% and 80 wt% concentrations. It was also concluded from these results that the fiber diameter tends to increase at higher polymer concentrations. No bead formation was observed for the 80 wt% PVP_10000_ concentration. Therefore, the optimized parameters to prepare bead-free fibers using PVP_10000_ were found to be 80 wt% concentration, 0.5 mL/h, and 20 kV applied voltage for 14 cm and 25G needle.

### 3.2. Parameter Optimization for PVP_55000_

**Figure 7:**
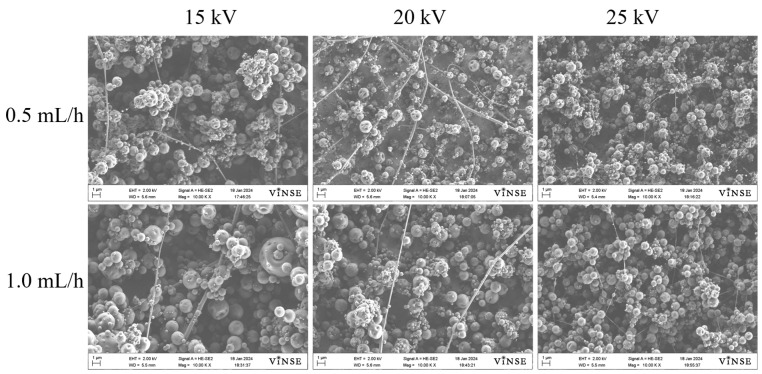
SEM micrographs of samples prepared with 30 wt% PVP_55000_ solution at 14 cm NCD.

As previously mentioned, spinning solutions with concentrations of 10 wt%, 30 wt%, and 50 wt% were prepared using PVP_55000_. The representative SEM micrographs for the 10 wt% PVP_55000_ concentration are displayed in Figure S2. No fiber formation was observed at any voltage or flow rate for this concentration due to the very low viscosity of the solution. In Figure 7, SEM micrographs of samples prepared using a 30 wt% PVP_55000_ solution at 15 kV, 20 kV, and 25 kV, with flow rates ranging from 0.5 mL/h to 1.0 mL/h, are shown.

**Figure 8:**
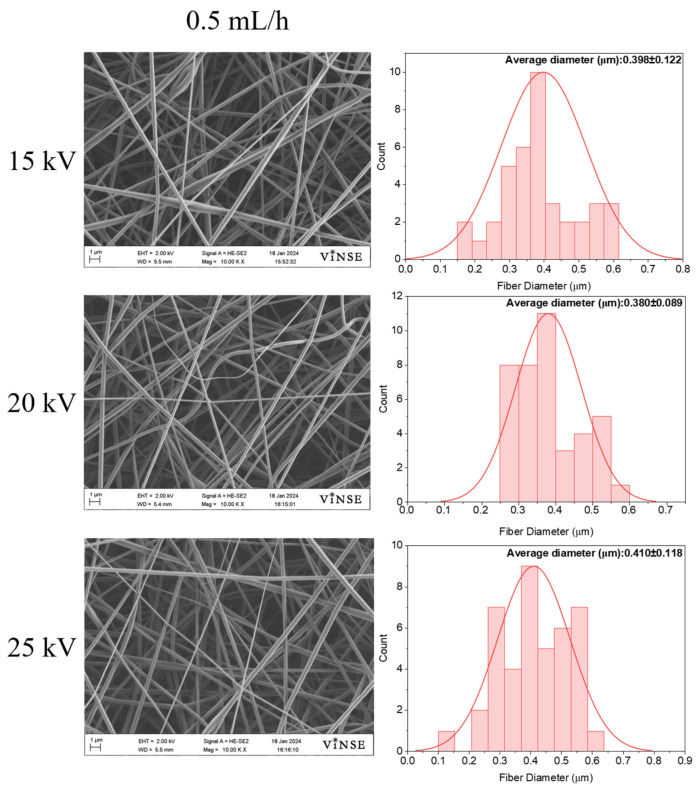
SEM micrographs of samples prepared using 50 wt% PVP_55000_ at 14 cm NCD and 0.5 mL/h flow rate.

Compared to the 10 wt% PVP_10000_ solution, these samples exhibited fiber fabrication; however, the particle density was still notably high in both cases. The fiber density for the 30 wt% PVP_55000_ solution was highest at a flow rate of 0.5 mL/h and 20 kV. Upon increasing the concentration to 50 wt%, a smooth fiber morphology was observed at a flow rate of 0.5 mL/h, as depicted in Figure 8.

Bead formation was observed at lower voltages (15 kV) but not at higher voltages. The average fiber diameters for a flow rate of 0.5 mL/h were found to be 0.398 ± 0.122 µm, 0.380 ± 0.089 µm, and 0.410 ± 0.118 µm for applied voltages of 15 kV, 20 kV, and 25 kV, respectively.

**Figure 9:**
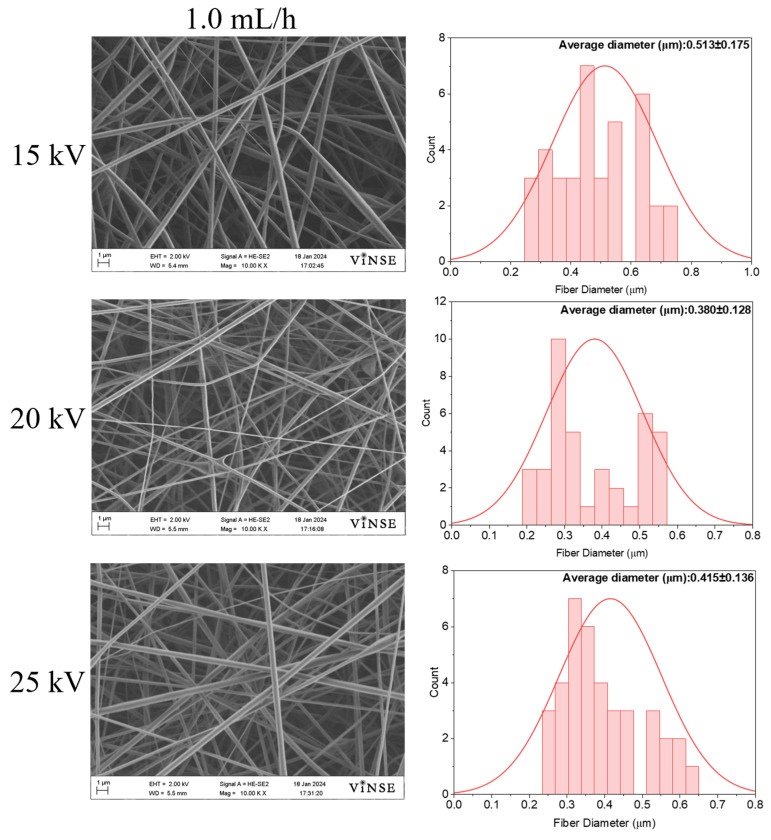
SEM micrographs of samples prepared using 50 wt% PVP_55000_ at 14 cm NCD and 1.0 mL/h flow rate.

To understand the effect of flow rate on fiber morphology, the flow rate was increased from 0.5 mL/h to 1.0 mL/h. Figure 9 shows the SEM micrographs and their corresponding diameter distributions for these samples.

**Figure 10:**
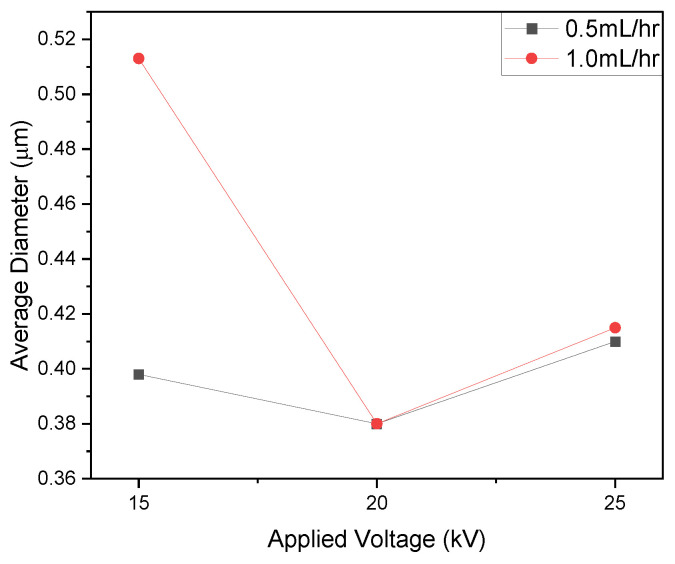
Graphical representation for the effect of flow rate on average fiber diameter; samples prepared using 50 wt% PVP_55000_, 14 cm NCD, and 25G needle.

The diameter of the fibers exhibited an increase when subjected to a higher flow rate of 1.0 mL/h at 15 kV, resulting in a measurement of 0.513 ± 0.175 µm. Additionally, the occurrence of bead formation was noted at the higher flow rate. The elevated flow rate causes the generation of larger droplets at the needle tip, with the charge applied to the droplets being insufficient to counteract the surface tension, thereby leading to the formation of beads. It tended to decrease at 20 kV applied voltage and increase at 25 kV (Figure 10).

The increase in polymer solution extraction at 25 kV, combined with a higher flow rate, resulted in greater bead formation at the higher voltage. Additionally, the diameter distribution was observed to increase with the flow rate. The optimized parameters for PVP_55000_ fiber fabrication were determined to be 50 wt% polymer concentration, 0.5 mL/h flow rate, and 20 kV applied voltage for 14 cm NCD and 25 gauge needle.

**Figure 11:**
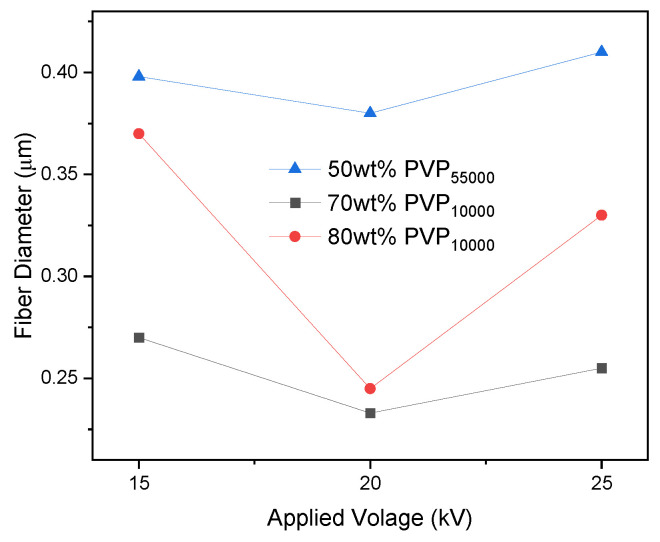
Graphical representation depicts variation in average diameter as a function of applied voltage for samples prepared at 0.5 mL/h using different concentrations (wt%) and PVP molecular weights.

Considering the SEM analysis of the fibers prepared by spinning 50 wt% solutions of both PVP_10000_ and PVP_55000_, it is concluded that a higher molecular weight significantly assisted in fiber fabrication. A molecular weight of 55,000 g/mol exceeds the critical entanglement molecular weight (generally in the range of 10,000–20,000 g/mol), which drastically increases the viscosity of the solution. Hence, fiber formation is observed for 50 wt% PVP_55000_; however, same is not true for PVP_10000_ [23]. However, increasing the concentration also leads to an increase in entanglement [24]; therefore, 70 wt% and 80 wt% PVP_10000_ exhibited fiber fabrication, as previously discussed. The fiber diameter of the samples prepared using 50 wt% PVP_55000_ was still higher. From detailed diameter analysis, it was also revealed that the higher molecular weight (55,000 g/mol in this case) assisted in the fabrication of fibers with higher diameters, even at lower concentrations. Figure 11 illustrates the fiber diameter plots at different concentrations of PVP_10000_ (70 wt% and 80 wt%) and PVP_55000_ (50 wt%).

### 3.3. Effect of Needle Gauge and NCD

As mentioned earlier, the needle gauge was changed to 22G for optimized electrospinning process parameters (deduced from the results discussed above) and solution concentrations of 80 wt% PVP_10000_ and 50 wt% PVP_55000_. The needle tip-to-collector distance (NCD) was varied between 12 cm and 16 cm to observe the variation in fiber morphology.

**Figure 12:**
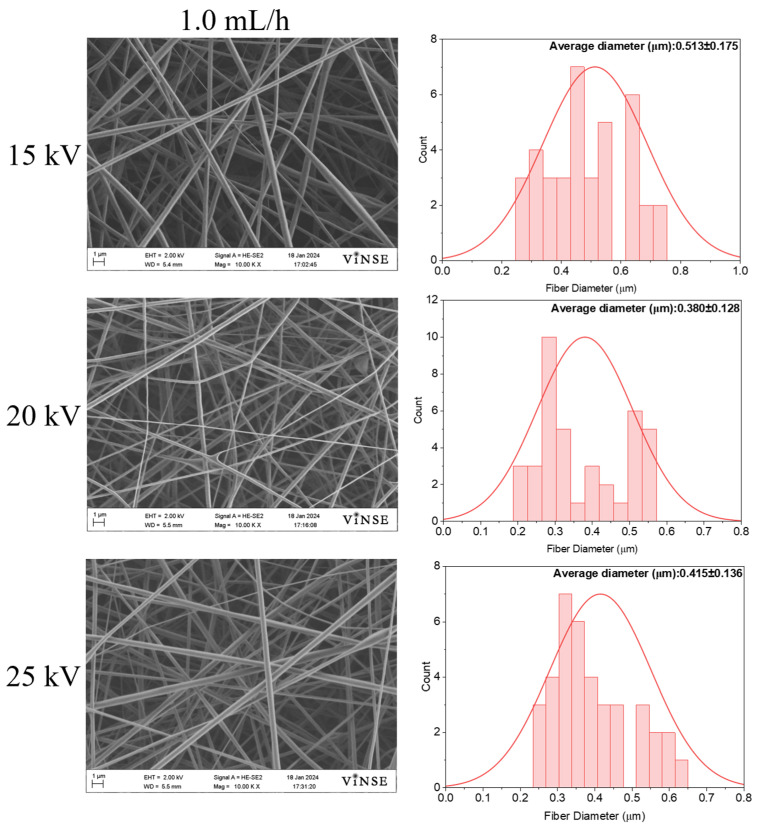
SEM micrographs of samples prepared using a 22G needle at 12 cm, 14 cm, and 16 cm at 20 kV, 0.5 mL/h, using 80 wt% PVP_10000_.

Figure 12 shows the SEM images and their corresponding diameter distributions for the samples prepared using 80 wt% PVP_10000_ at three NCDs (12 cm, 14 cm, and 16 cm) at 20 kV and 0.5 mL/h, using a 22G needle.

Average diameters were observed to be 0.349 ± 0.118 µm, 0.297 ± 0.089 µm, and 0.341 ± 0.091 µm for 12 cm, 14 cm, and 16 cm NCD, respectively. It was observed that by increasing the distance from 12 cm to 14 cm, the fiber diameter tended to decrease. This trend is associated with the stretching of the fiber jet for longer NCD, which led to the decrease in the fiber diameter. However, at 16 cm, the fiber diameter increased to 0.341 ± 0.091 µm, indicating that the electrostatic force due to the applied voltage (20 kV) for this distance did not suffice to stretch the jet enough to decrease the fiber diameter [25,26]. No bead formation was observed at any distance. The diameter distribution became narrower with an increase in distance, indicating a better uniformity of the fiber diameter. The most uniform fiber diameter deposition was observed for samples prepared at 14 cm. As compared to fibers fabricated using identical process parameters for 25G and 14 cm NCD, the fiber diameter was observed to increase when a 22G needle was used. This increase was attributed to the larger drop formed at the needle tip due to the increased needle diameter. The applied voltage was high enough to create a charge that overcame surface tension, along with the optimal viscoelasticity of the polymer solution, preventing bead formation.

**Figure 13:**
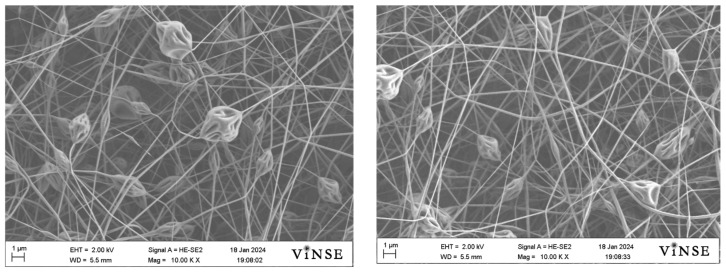
Representative SEM micrographs of samples prepared using a 22G needle, 0.5 mL/h, 20 kV with 50 wt% PVP_55000_.

When the PVP_55000_ solution was used for electrospinning using a 22G needle at different distances (12 cm, 14 cm, and 16 cm), a significantly high bead density was observed. The variation in distance or lower flow rate for this concentration did not assist in decreasing the bead formation. This occurred due to the wider diameter of the 22G needle (0.413 mm). The larger drop formation resisted the applied voltage to overcome the surface tension, which led to the formation of large beads. Given that the spinning solution demonstrated no bead density when electrospun with a 25G (0.260 mm) needle, it can be inferred that the solution parameters were suitable for this needle gauge. However, in order to prevent bead formation when utilizing a 22G needle, additional optimization of the solution concentration is necessary. It worth mentioning that the same results were observed for all the distances; therefore, the representative SEM micrographs for these samples have been shown in Figure 13. Increasing the concentration of PVP_55000_ may lead to a decrease in bead formation when using a smaller needle gauge.

## 4. Conclusions

This study successfully optimized the process parameters for producing electrospun fibers of PVP with lower molecular weights of 10,000 g/mol and 55,000 g/mol. It was found that a polymer with a lower molecular weight necessitates a higher polymer concentration to achieve the optimal viscosity for fiber production. Fiber ejection depends significantly on the higher viscoelasticity of the solution; therefore, preparation of the solution is a crucial step to prepare smooth, bead-free fibers. Moreover, it was observed that an increase in flow rate can lead to an increase in fiber diameter; however, the influence of flow rate at higher voltages is negligible. If the viscoelasticity of the solution and the applied charge density on the solution drop are not high enough to overcome the surface tension, bead formation is observed, especially at a higher flow rate, as observed for 50 wt% PVP_55000_. Same observation was made when the needle gauge was reduced (the inner diameter of the needle increased). To prepare thinner fibers, a lower molecular weight is observed to be ideal. There are some polymers that hold great potential for drug delivery; however, their lower molecular weights hinder the fiber formation. The main objective of this research was to enhance the electrospinning process conditions in order to produce smooth bead-free fibers using a polymer with a lower molecular weight. It can be inferred that this objective has been successfully met. Another aim of this study was to explore the underlying principles of the electrospinning process variables that influence fiber morphology. This research effectively examined the combined and individual impacts of various electrospinning process parameters on the fiber structure. The findings of this study will aid in comprehending the basic principles necessary to optimize process parameters for the creation of fibers from these types of polymers.

## 5. Future Work

A thorough investigation will be conducted to analyze and correlate the viscosity of the spinning solution (as a function of concentration and molecular weight) in a systematic manner. To further investigate how different electrospinning process parameters impact composite formation, the plan is to encapsulate carbon nanotubes (CNTs) within PVP fibers using a uniaxial electrospinning system. The study will aim to assess the potential of CNTs embedded in PVP fibers for microwave absorption applications. Additionally, the impact of using higher voltages (>25 kV) in producing PVP fibers and CNTs/PVP composite fibers will be examined to understand how voltage affects their morphology and, consequently, their transport properties (such as electrical and thermal characteristics). This fundamental research will also assist in optimizing process parameters for other polymers (like poly vinyl alcohol) with lower molecular weights that will be utilized in future studies.

## Figures and Tables

**Table 1:** Electrospinning process parameters used for different concentrations of PVP_10000_ and PVP_55000_.

Process Parameters	10 wt%	30 wt%	50 wt%
Needle Gauge (G)	25 (inner diameter: 0.260 mm)	25 (inner diameter: 0.260 mm)	25 (inner diameter: 0.260 mm)
NCD (cm)	14	14	14
Voltage (kV)	15, 20, 25	15, 20, 25	15, 20, 25
Flow rate (mL/h)	0.5, 1.0 (both flow rates at each voltage)	0.5, 1.0 (both flow rates at each voltage)	0.5, 1.0 (both flow rates at each)

**Table 2:** Electrospinning process parameters used to study the effect of needle gauge and needle-to-collector distance (NCD) on fiber morphology.

Solution wt%	Needle Gauge (G)	NCD (cm)	Voltage (kV)	Flow Rate (mL/h)
PVP_55000_ 50 wt%	22 (ID: 0.413 mm)	12, 14, 16	20	0.5
PVP_10000_ 80 wt%	22 (ID: 0.413 mm)	12, 14, 16	20	0.5

## Data Availability

Data are contained within the article and Supplementary Materials.

## Supplementary Materials

The following supporting information can be downloaded at https://www.mdpi.com/article/10.3390/polym16091217/s1, Figure S1: Representative SEM micrographs of the samples prepared using 10 wt% spinning solution PVP_10000_. Figure S2: Representative SEM micrographs of the samples prepared using 10 wt% spinning solution PVP_55000_.
