# Supplementary material for: Optimization of Electrospinning Parameters for Lower Molecular Weight Polymers: A Case Study on Polyvinylpyrrolidone
Source: Polymers (Basel). 2024 Apr 26;16(9):1217. doi: 10.3390/polym16091217 (PMC11085657; doi:10.3390/polym16091217)
Supplement: Supplementary file 1 [file polymers-16-01217-s001.zip › polymers-2963469-supplementary.pdf]

Supplementary Information

Figures below show the representative SEM micrographs of the samples prepared at different electrospinning conditions using PVP_{10000} and PVP_{55000}.

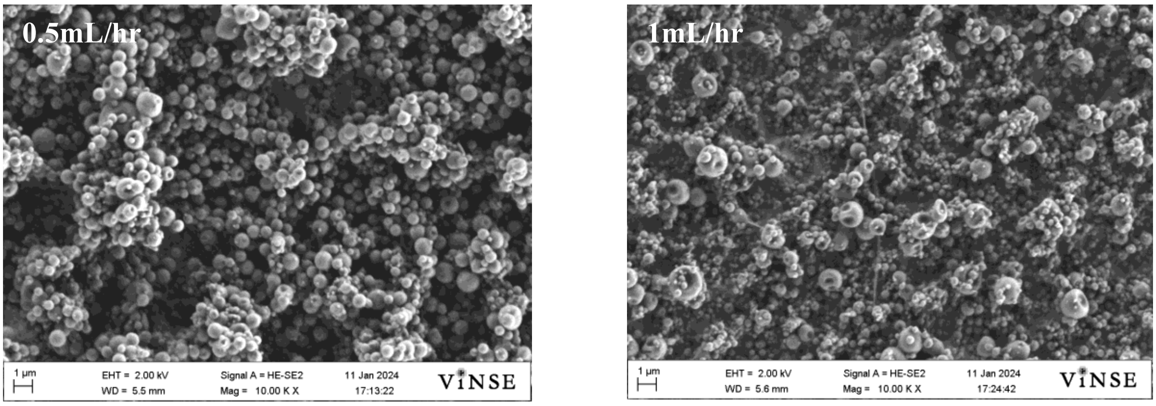

Figure S1. Representative SEM micrographs of the samples prepared using 10wt% spinning solution PVP_{10000}.

The particle formation was observed for all the samples at different sets of electrospinning process parameters.

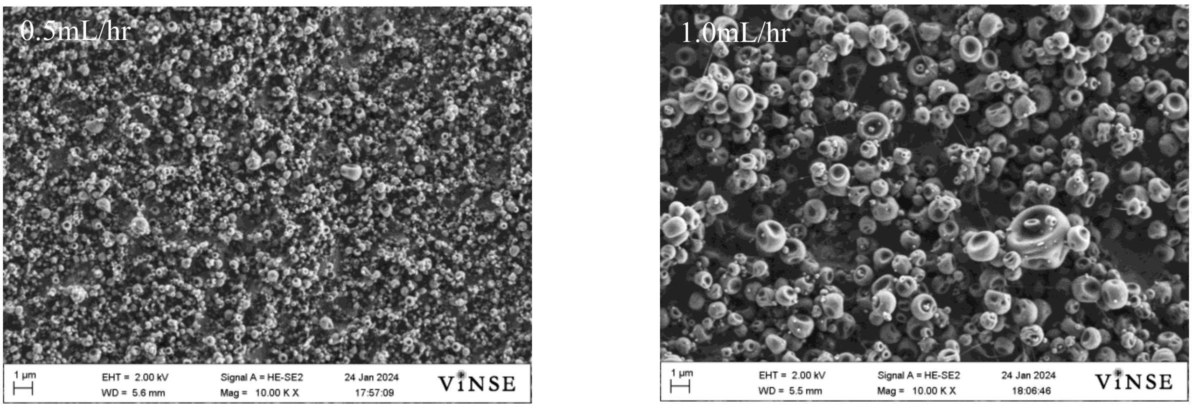

Figure S2. Representative SEM micrographs of the samples prepared using 10wt% spinning solution PVP_{55000}.

For all the samples prepared using 10wt% spinning solution of PVP_{55000} showed particle formation. Variation in flow rate, applied voltage, needle to collector distance and needle gauge did not show fiber fabrication and showed particle formation for all sets of process parameters.
